# *In house* ELISA based on recombinant ORF2 protein underline high prevalence of IgG anti-hepatitis E virus amongst blood donors in south Brazil

**DOI:** 10.1371/journal.pone.0176409

**Published:** 2017-05-09

**Authors:** Rafael Pandolfi, Denise Ramos de Almeida, Marcelo Alves Pinto, Luiz Carlos Kreutz, Rafael Frandoloso

**Affiliations:** 1 Laboratório de Microbiologia e Imunologia Avançada, Faculdade de Agronomia e Medicina Veterinária, Universidade de Passo Fundo, Passo Fundo, Rio Grande do Sul, Brazil; 2 Laboratório de Desenvolvimento Tecnológico em Virologia/IOC - Fundação Instituto Oswaldo Cruz, Rio de Janeiro, Rio de Janeiro, Brazil; Centers for Disease Control and Prevention, UNITED STATES

## Abstract

Hepatitis E Virus (HEV) is a zoonotic pathogen responsible for causing acute hepatitis in human, especially in developing countries. Diagnosis of HEV usually relies on the detection of antibodies mostly by enzyme-linked immunosorbent assay (ELISA). In the present study, we designed a new indirect ELISA (iELISA) based on a short recombinant peptide derived from the capsid protein (ORF2p) and demonstrated its potential for detecting human IgG against HEV genotype 3. The best polystyrene plate (Maxisorp^®^), optimal ORF2p coating antigen concentration (0,67μg/well) and primary antibody dilution (1:100) were determined. This iELISA showed a sensitivity of 91.4% and specificity of 95.9%. The comparison of our *in house* iELISA with a commercial assay (RecomWell, Mikrogen^®^) showed 94.25% of agreement and a *kappa* index of 0.88. The ORF2 recombinant ELISA was used to screen 780 blood donors for anti-HEV IgG and we found that 314 (40,25%) of these donors were IgG positive. This high prevalence of antibodies suggests, for the first time, that the Southern Brazil region might be endemic to Hepatitis E Virus genotype 3.

## Introduction

Viral hepatitis stands up as a major public health issue worldwide and is caused by several different types of enterically and parenterally transmitted viruses. Hepatitis E (HE), for instance, caused by hepatitis E virus (HEV) is endemic in several developing African, Asian and South American countries [1] and autochthones cases are found at increasing and steadily frequency in developed countries [2]. The infection by HEV is usually unnoticed except in pregnant women and patients with liver-related problems [3]. However, a recent report indicated that the infection might become chronic mainly in immunocompromised individual such as kidney and liver-transplanted [4] and HIV-positive subjects [5]. In this scenario, one of the major question relates to the role HEV might take in causing chronic hepatitis in individual under immunosuppressive medication treatments [6] and recipients of blood derived product [7]. Thus, screening for anti-HEV antibodies or HEV RNA amongst blood donor should become mandatory.

HEV is a small icosahedral non-enveloped RNA virus with a single-stranded positive-sense genome with approximately 7.3 kilobases classified in the genus *Hepevirus*, *Hepeviridae* family [8]. The viral genome contains three open reading frames (ORFs) that encode the structural and nonstructural proteins. There are 4 well-known genotypes with distinct epidemiological distributions: genotypes 1 and 2 are epidemically found in Asia, Central and South America and in some African countries [9] and infect exclusively humans [10] whereas genotypes 3 and 4 are found mostly in Asia and developing countries and might cause infection in humans and animals, mainly domestic pigs [11] and wild boars [12]. Although there is a strong evidence of cross species transmission infection between human and pigs [13] the role of other animal species in the epidemiology of HEV infection remains to be evaluated.

In endemic countries the transmission of HEV occurs mostly by the oral-fecal route [14]; conversely, in developed countries, foodborne transmission [15], blood transfusion [7] and transplants of solid organs such as heart, lung, liver and kidney [16] are becoming major source of viral dissemination. The ingestion of undercooked contaminated pork meat and pork-related product might also constitute a potential source of infection [17]. However, the scarcity of data on these routes of infection hampers further evaluation on HEV epidemiology and the impact on public health. Nonetheless, the detection of anti-HEV antibodies or HEV nuclei acid amongst blood donors [18, 19] indicates that viral spread might be long occurring and the prevalence and incidence of HEV might be even higher than previously thought. In Brazil, HEV genotype 3 is commonly found in pig farms [20] and autochthonous human cases of HEV have already been described here [21], in Germany [22], France [23], Cambodia [24] and Israel [25] strengthening the zoonotic potential of HEV.

HE diagnosis is based mostly on the detection of anti-HEV antibodies (IgM, IgG and IgA) towards ORF-2 and ORF-3 encoded proteins [26]. Currently, there are commercial kits available to detect anti-HEV antibodies that differ in specificity and sensibility, mainly when used to HEV diagnosis in non-endemic countries [26, 27]. Recently, we expressed and characterized a recombinant protein based on the C-terminal of ORF-2 protein (ORF2p) from HEV genotype 3 and demonstrated its antigenic and diagnosis potential [28]. Here, we developed an indirect ELISA based on the recombinant ORF2p and screened blood samples from healthy blood donors to evaluate the prevalence of anti-HEV antibodies. We found a high prevalence of positive samples which indicates that the region might be potentially endemic to HEV.

## Material and methods

### Expression and purification of ORF2 recombinant protein

The recombinant HEV genotype 3 capsid protein was produced as previously described by our group [28]. Briefly, the plasmid pET20-His-Mbp-TEV-ORF2p was transformed into competent *E*. *coli* strain ER 2566 and induced with 100 mM isopropyl-β-D-thiogalactopyranoside (IPTG, Sigma). Culture media was centrifuged (8,000 × g, 1 h, 4 °C) and the bacteria pellet was suspended in NTA buffer (20mM NaH_2_PO_4_, 500 mM NaCl, 20 mM Imidazole, pH 8.0) and sonicated three times at 70 watts (Ultronique QR, Eco-sonics, Brazil). The bacteria lysate was then centrifuged (13.000 × g, 1 h, 4°C) and the supernatant was filtered (0.22 μM) to purify the protein by a two-step procedure, using the ÄKTA Pure^®^ chromatography system (GE Healthcare, Germany) with HisTrap and Sepharose Q columns connected (both from GE Healthcare). The ORF2p was dialyzed in PBS and kept at -80°C until use.

### Blood samples

The number of samples required for this study was calculated considering a prevalence of anti-HEV antibodies of 10%, a confidence interval of 95% and a precision of 2%. Then, seven hundred and eighty (780) samples were selected from the blood bank (Hemocentro de Passo Fundo—Hemopasso), in the state of Rio Grande do Sul, from March to October 2015. All samples were from healthy and suitable blood donors as required by the law number 1.075 from the Brazilian Ministry of Health. The study was approved by the Institutional Committee on Research Ethics (process number 849709).

### ELISA assay design

Two commercially available polystyrene microplates (Maxisorp^®^ and Polysorp^®^, Nunc, USA) were evaluated regarding the ability to adsorb the ORF2p. A total of 5 positive and 5 negative serum samples, previously evaluated by a commercial ELISA (RecomWell anti-HEV IgG, Mikrogen^®^, Germany) were tested in triplicates in three different plates. The plates were coated with ORF2p (5 μg/well) diluted in carbonate buffer (pH 9.6) at 4°C for 12 h and then washed three times with phosphate buffered saline 0.05% Tween (PBST, pH 7.2). Plate wells were blocked with PBST containing 3% bovine serum albumin (BSA) at 37°C for 2 h. Human serum diluted 1:100 in PBST 1% BSA was added and allowed to react for 1 h at 37°C. After washing, peroxidase conjugated goat anti-human IgG (Santa Cruz Biotecnology^®^, USA) diluted 1:5,000 in PBST 1% BSA was added and the plates were incubated as indicated above, followed by three washes and addition of substrate (3,3, 5,5’-tetrametilbenzidina + 0,06% H_2_O_2_). The plates were then incubated in the dark at 22 °C for 10 min and the reaction was stopped by adding 3 N HCl. Plates were read at 450 nm using a Synergy HI plate reader (BioTek^®^, USA).

The mean positive/negative (P/N) ratio and the mean within-plate percent coefficient of variation (CV%) were calculated for each type of plate as previously described [29]. The plates were then compared using an index obtained by dividing the P/N ratio by the CV%.

The optimal concentration of antigen was determined by titration in which twofold antigen dilution (final volume of 100 μl) was added to the plates and titrated against the positive and negative sera. The optimal antigen concentration was defined as the lowest antigen concentration that caused no significant changes in the OD obtained with the positive and negative sera.

Eight dilutions of the positive and negative serum samples were used to determine the ideal dilution to be used in the iELISA. Serum samples were diluted 1:100 to 1:1.000 in PBS in a 96-DeepWell™ plate (Nunc^®^, USA) and then 100μl of each dilution was transferred in duplicate to the sensitized plate wells (Maxisorp^®^, Nunc, USA). The optimal dilution was defined taken in consideration the mean absorption and the serum dilution as recommended previously [30]. Two diluents, PBST 1% BSA and PBST 1% skin milk were also analyzed.

### Cut-off point determination

The iELISA cut-off point was set considering the relation between the optical density (OD) of the sample (OD_S_) and the OD of the negative control (OD_NC_). The OD_NC_ was calculated using 50 serum samples that were free of anti-HEV IgG and IgM as determined by a commercial ELISA (RecomWell anti-HEV IgM and IgG (Mikrogen^®^, Germany) and free of HEV RNA [28]. Also, a total of 63 positive serum samples (anti-HEV IgG, determined by RecomWell kit) were included for the cut-off determination. The Receiver Operating Characteristic (ROC) curve analysis was performed to set the threshold taking into account the specificity and sensitivity of this new iELISA. The Area Under the Curve (AUC) analysis was used to evaluate the ability of this test to discriminate between those individuals that have IgG against HEV genotype 3 and those without any IgG antibody against the virus. The sample was considered positive when the relation OD_S_/OD_NC_ was higher than 2.5.

### Statistical analysis

The Kolmogorov-Smirnov test was used to determine the normal distribution of the data. The results were analyzed by Kruskal-Wallis or One Way Anova followed by Tukey post-test according to the data. Significant differences were considered when p < 0.05. All the statistics were performed using the GraphPad Prism software (GraphPad, USA).

## Results

### Microplates

**Fig 1:**
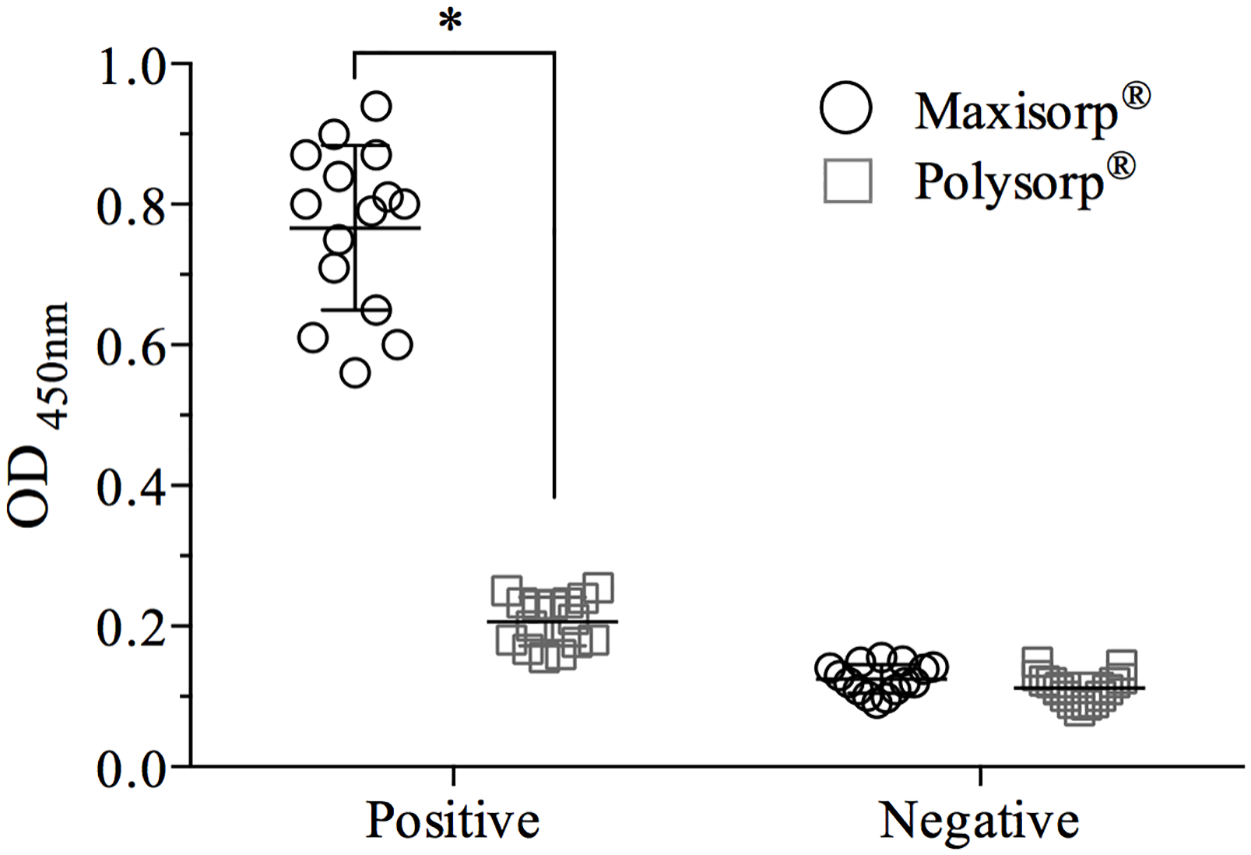
Performance of ELISA Maxisorp^®^ and Polysorp^®^ microplates in the in house ELISA assay. Plates were sensitized with recombinant capsid protein (5 μg/well) and reacted with 5 positive and 5 negative samples previously tested with the commercial kit. Significant differences (p<0.05) on the OD obtained with the same set of serum are indicated by asterisk (*).

The Maxisorp^®^ microplate adsorbed recombinant ORF2p more efficiently than the Polysorp^®^ microplate. The mean OD of the positive control samples (0.76 ± 0.11) was 3.7 times higher (p = 0.0011) in the Maxisorp^®^ plate (Fig 1). The mean of the OD_NC_ were similar amongst plates (0.125 ± 0.021 in Maxisorp^®^ and 0.121 ± 0.034 in Polysorp^®^ microplate). The adsorption of ORF2p to the Maxisorp^®^ was more constant (lower CV%) and allowed a better distinction between positive and negative samples (P/N ratio) compared to the Polysorp^®^. The P/N value normalized with its CV% obtained in the Maxisorp^®^ microplate (1.24) was 4.27 times higher than that observed in the Polysorp^®^ microplate (0.29) (Table 1).

**Table 1:** Performance of ELISA microplates. Plates were sensitized with recombinant ORF-2 protein and tested with known positive (P) and negative (N) serum to determine the P/N relation and the percentile of the coefficient of variation (CV%). The index value was obtained by dividing the result of P/N by the CV%.

Brand	Reference	P/N	% CV^a^	Index^b^
**Nunc**	Maxisorp^®^	7,07	5,7	1,24
**Nunc**	Polysorp^®^	2,16	7,4	0,29

^*a*^ Percentile of the coefficient of variation (CV) from positive samples tested in triplicates in each microplate.

^*b*^ Index obtained by dividing the P/N ratio by the CV%. The microplates were classified by this index.

### Optimal antigen concentration and primary serum dilution

**Fig 2:**
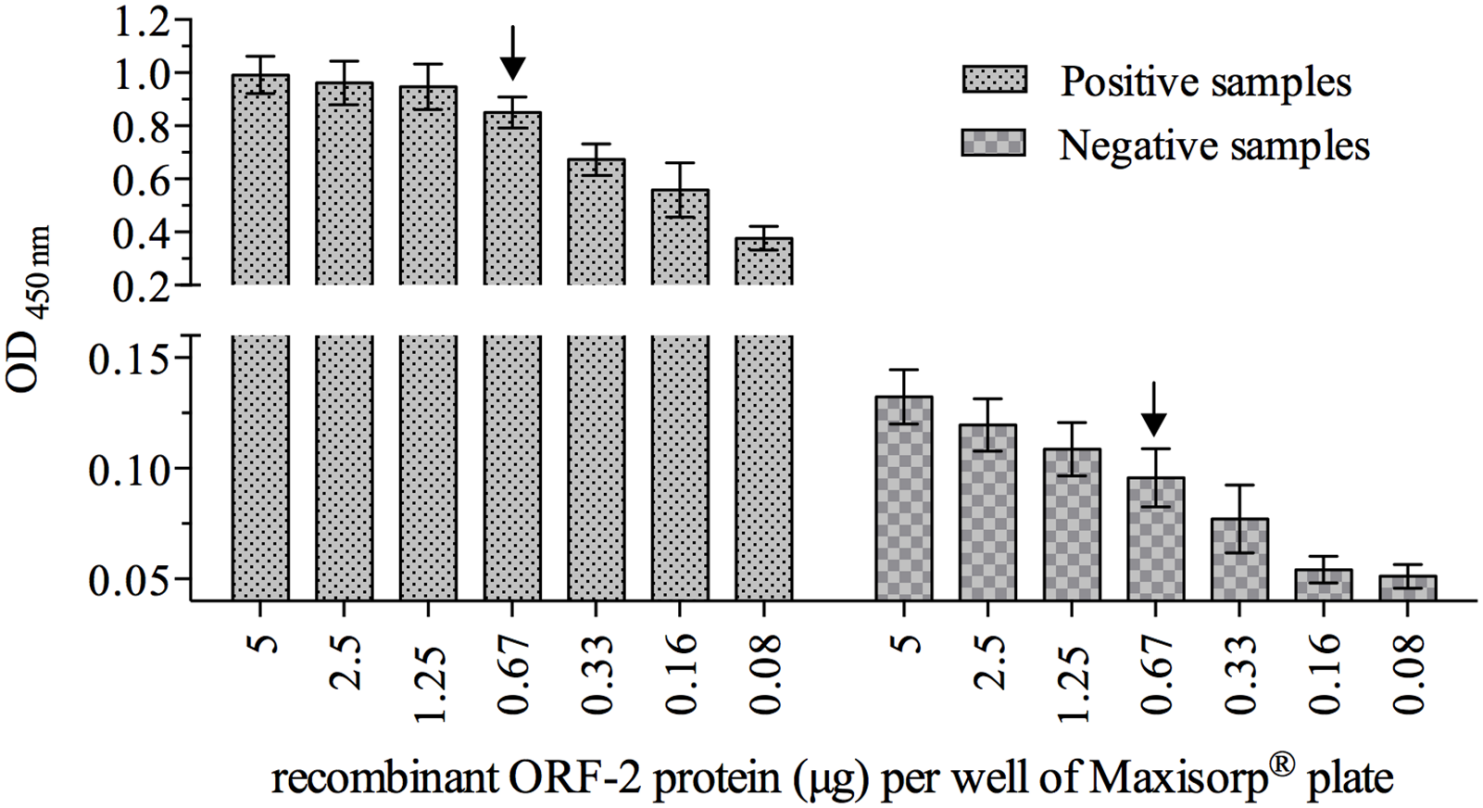
Optimal antigen concentration for the in house ELISA. Recombinant capsid protein (5 μg) was twofold serially diluted on Maxisorp^®^ microplate. Serum samples, previously determined to be positive (n = 5) or negative (n = 5) were evaluated. The data is represented as the mean ± SEM of the OD of each antigen dilution. The arrow indicates the optimal antigen concentration.

The ideal concentration of ORF2p was determined by twofold serial dilution using the Maxisorp^®^ microplate and was adjusted at 0.67 μg/well (Fig 2). At this antigen concentration the mean OD readings obtained from the positive and negative control samples were similar to that obtained at the highest antigen concentration (5 μg/well) but significantly different (p = 0.0008) from the next concentration (0.33 μg/well). The remaining experiments were then performed using the ORF2p at 0.67 μg/well.

**Fig 3:**
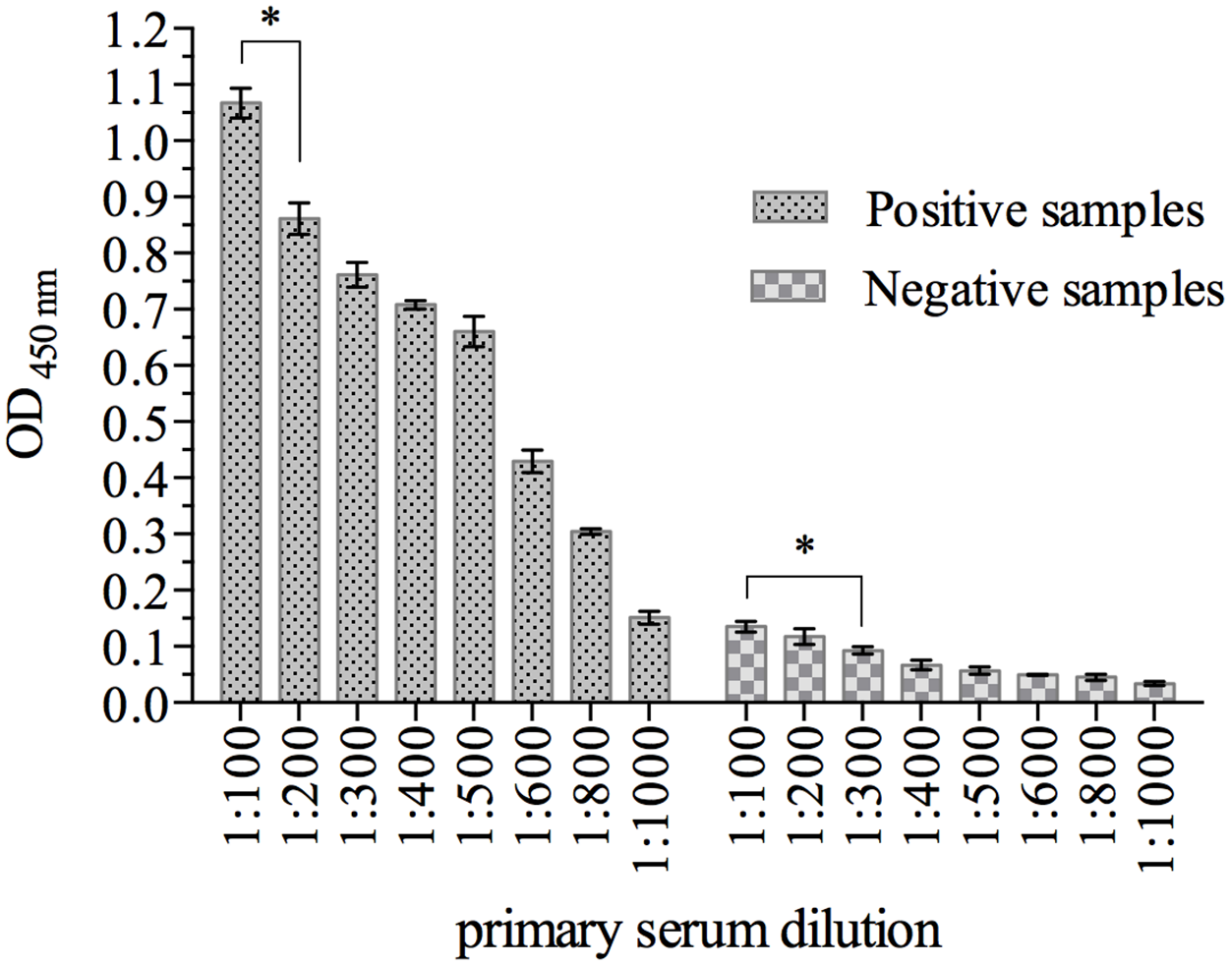
Determination of the primary serum dilution. Human serum samples known to be positive (n = 5) or negative (n = 5) were diluted as indicated in the figure and tested for their ability to bind the recombinant capsid protein (5 μg/well) adsorbed onto Maxisorp^®^ ELISA microplates. The results are expressed as the mean ± SEM of the OD obtained at each serum dilution. The asterisk (*) indicates the lowest serum dilution with significant difference (p<0.05) to the immediately higher dilution within positive or negative samples.

The optimal dilution of human sera to be used in the iELISA was determined taken into consideration the P/N OD relation. Positive control samples diluted 1:100 gave a significantly higher (p = 0.0001) OD reading mean than that obtained at the 1:200 dilution (Fig 3). At the same dilution (1:100), the mean OD of the negative control samples was below 0.15 and was similar at the 1:200 and 1:300 dilutions. A significantly (p = 0.0001) lower OD mean was obtained only when negative control samples were diluted more than 1:300. Thus, in the remaining experiments serum samples were diluted 1:100 to be evaluated.

### Specificity and sensitivity of iELISA

**Fig 4:**
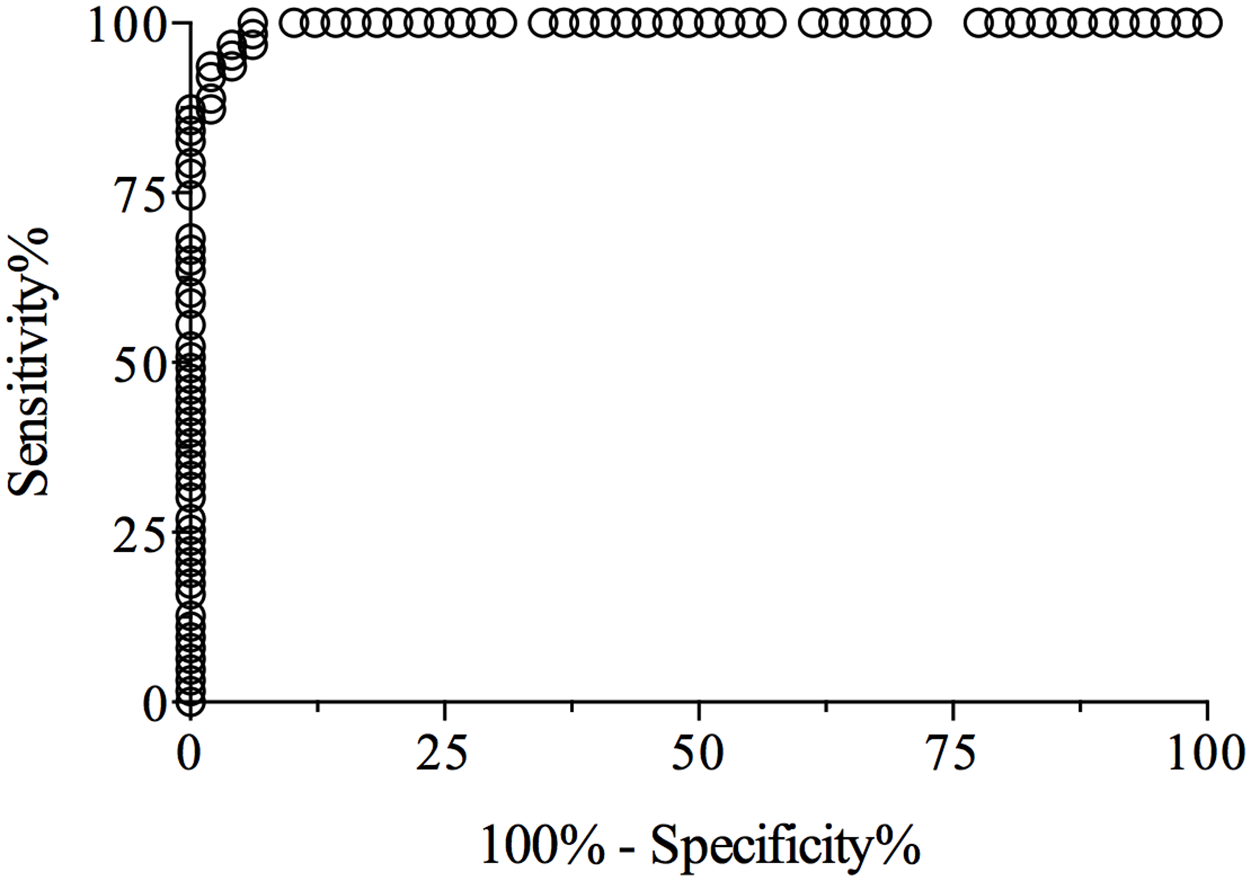
Receiver Operating Characteristic (ROC) analysis. The ROC curve was generated using the results obtained by analyzing 50 negative human serum samples and 63 positive human serum samples by the iELISA. The area under the ROC curve was of 0.9955.

The cut-off value, specificity and sensitivity of the iELISA were determined by testing 50 negative and 63 positive human serum samples for antibodies against HEV. The ROC curve analysis of the iELISA data produced paired estimates of relative sensitivity and relative specificity at different cut-off values. A cut-off of OD ≥ 0.31 was recommended. At this cut-off value, the relative sensitivity and specificity estimates were 96.8% (95% confidence interval = 91.4% to 99.6%) and 95.9% (95% confidence interval = 88.7% to 99.5%), respectively. The Likelihood ratio at this cut-off was 42.78. The ROC curve (Fig 4) had an AUC value of 0.9955 (95% confidence interval = 0.988 to 1.002), which indicated a high level of accuracy for this iELISA. Based in this cut-off value we generated a relation between OD_S (sample)_ and OD_NC (negative control)_, and the sample was considered positive when the relation OD_S_/OD_NC_ was higher than 2.5, as previously suggested [31].

### Comparison between *in house* iELISA and commercial assay

The performance of the *in house* iELISA to detect anti-HEV IgG was compared with the commercial ELISA assay (RecomWell, Mikrogen^®^, Germany). We randomly selected 87 samples previously tested in the *in house* iELISA and evaluated them in duplicates using the commercial ELISA. The sample panel was composed by 49 positive and 38 negative sera. When analyzed in the commercial ELISA we found 50 positive and 37 negative samples (Table 2) resulting in 94,25% of agreement and a *Kappa* (*K*) index of 0.88 (strong/very good agreement).

**Table 2:** Agreement between the performance of the in house ELISA and the RecomWell (Mikrogen^®^) in detecting anti-HEV IgG. Eight seven serum samples were randomly selected amongst the samples used in this study and tested in duplicates with the commercial ELISA kit to determine the presence of anti-HEV antibodies.

		Commercial ELISA kit		
		Positive	Negative	Total
***in house iELISA***	**Positive**	47	2	49
	**Negative**	3	35	38
	**Total**	50	37	87

### Prevalence of anti-HEV IgG in blood donors

**Fig 5:**
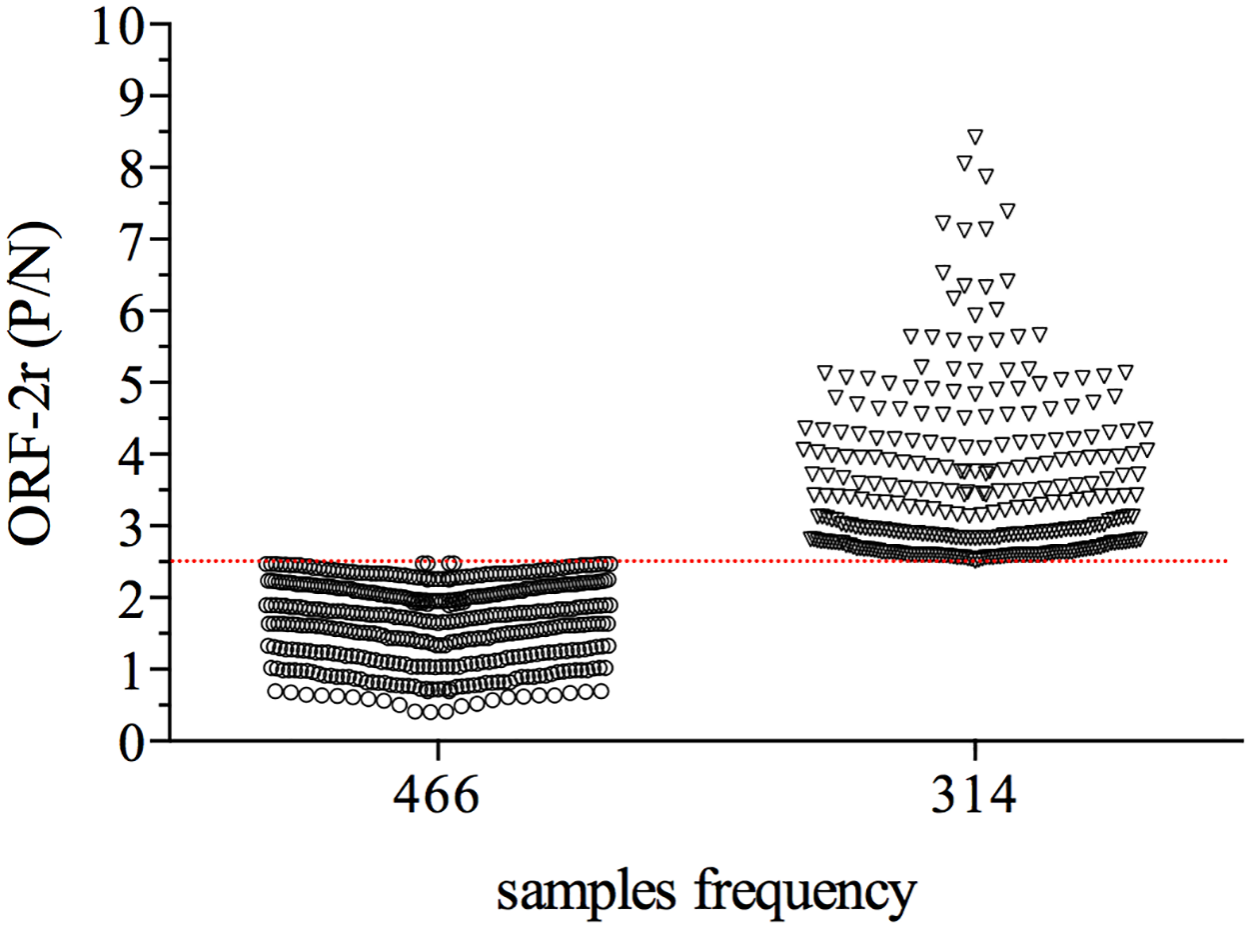
Prevalence of anti-HEV antibodies amongst blood donors. The in house ELISA was performed using the ideal concentration of antigen and ideal serum dilution, with 780 blood samples. The results are expressed as the P/N ratio, as described in material and methods. Samples with a P/N ratio was ≥ 2.5. The cut-off value is represented by the dashed line.

Aiming to evaluate the prevalence of anti-HEV IgG amongst blood donors we analyzed 780 samples collected from March to October (2015) at a major blood bank from north region of the Rio Grande do Sul State. We found that 314 (40.25%) samples had OD reading higher than the *cut-off* value that we set for the assay (Fig 5) and where thus considered positive to the presence of anti-HEV IgG antibodies. Amongst the positive samples, 90% had OD readings 2.5 to 5 times higher than the average obtained with the negative control samples and the remaining 10% of the positive samples had a P/N ratio higher than 5.

## Discussion

Hepatitis E is a neglected disease in Brazil and most developing countries and fatalities rates are of major concern to health authorities [31]. The course of HEV is usually asymptomatic and short lived [32] but in immunocompromised patients HEV might persist causing chronic infection and hepatic failure, neurological syndrome, renal injury and autoimmune hepatitis [32]. Furthermore, because HEV might be transmitted through blood transfusion [7], screening for anti-HEV antibodies in blood donors should help in preventing widespread dissemination of the virus, mainly in industrialized countries.

Recently, we described the production of a potential candidate antigen for the development of diagnosis to HEV [28]. The viral capsid protein, encoded by ORF 2 gene, is highly immunogenic and widely used in immunosorbent assays to detect anti-HEV antibodies in humans and animals [33–36]. Although different genotypes of HEV share common epitope within the ORF2 protein [37, 38], a better immunoreactivity in the ELISA platform is achieved when the antigens used to coat the plate belong to the same HEV genotype that is infecting the patients or animals tested [39]. Here, we describe the development of an indirect ELISA assay using a recombinant viral capsid protein suitable to detect anti-HEV antibodies in blood donors. We found that the characteristics of the microplate can affect the outcome of the assay. The MaxiSorp^®^ microplate had a higher (p = 0.0011) antigen binding efficiency and a lower (p = 0.0021) CV compared to PolySorp^®^ microplate. The MaxiSorp^®^ microplate adsorbs molecules with hydrophilic and hydrophobic domains whereas PolySorp^®^ microplates are more suitable to adsorb molecules with hydrophobic domains.

The optimal concentration of antigen was set at 0.67 μg/well; considering the maximal binding capacity/cm^2^ (0.5 μg of protein) and the final volume used in the assay (100 μl), the estimated saturation would occur with 0.42 μg of antigen. Indeed, in our study we found that with an antigen concentration of 0.33 μg/well the average OD value obtained with the positive control was significantly (p = 0.0008) lower compared to a higher antigen concentration (0.67 μg/well). The optimal antigen concentration in our study is higher than that reported previously [34] in which 0.25 μg/well of antigen was used. In similar studies, even lower antigen concentrations (15 ng and 30 ng/well) have been used in indirect ELISA to HEV [35, 38]. Considering that all studies evaluated the best microplate to be used in the assay, the differences in the amount of antigen used might be attributed to the system in which the recombinant protein was produced and with the size of the antigen used, that varied from approximately 30 KDa [28, 34] to 68 KDa [35, 38]. When antigens containing only the C-terminal domain of ORF2p are used, as reported here and by others [34], a higher antigen concentration is needed to obtain OD reading similar to that obtained when the entire ORF2p is used, indicating that the N-terminal domain of the protein contains important antigenic epitopes that should be useful for serological diagnosis. Furthermore, the ideal dilution of the primary antibody (1:100) in which the negative control samples had OD reading lower than 0.15, is similar to that reported with other iELISA assays for HEV [34, 35, 38].

The performance of our iELISA assay was compared to a commercial assay (RecomWell^®^) which is known for its higher sensitivity and widely use in epidemiological studies in areas endemic for HEV genotype 3 [40]. We found 94.25% of agreement between the results and a K value of 0.88, similar to the results reported previously by Jímenez de Oya (35) and slightly better when compared to the study published by Arankalle (36). It is worth noticing that the antigen we used was expressed in *E*. *coli* rather that in eukaryotic cells [35, 36] but still we obtained a high quality antigen suitable to be used in immunoassays.

In Brazil, antibodies to HEV genotype 3 were detected in pigs farms [20, 41, 42] and HEV was found in a case of human hepatitis [21] but blood related products were not evaluated yet for the presence of HEV RNA and limited is the information about the antibodies prevalence. In addition, because of the recent transfusion-transmitted HEV infection, blood products might pose a risk to immunocompromised or organ transplanted patient [40]. Thus, using our iELISA, we analyzed 780 blood samples from a major blood bank. Blood donors were from the North region of Rio Grande do Sul state, a region with a high density of pigs farms and high consumption of pig meat and related products. Amongst the samples analyzed, 314 (40.25%) had antibodies to HEV, a prevalence much higher than that reported recently in a neighboring state [43] or other countries. Even though differences in the ELISA settings and sensitivity of the assay might account for differences in the percentile of seropositive found amongst different studies [44] we credit the higher percentile of seropositive samples to characteristics inherent to the population studied and to the geographical localization. Endemic areas to HEV genotype 3 are found in France [19] and Cambodia [24] in which the prevalence of IgG antibodies to HEV was 52.5% and 28.5% amongst blood donors respectively. The population of our study resides within a region with high density of pig farms in which 15% of Brazilian pigs are produced [45]. Furthermore, more than 80% of the blood donors indicated that pig meat or related product are part of their everyday diet (data not shown) and this could account for the higher prevalence of anti-HEV antibodies, as already reported in other countries [19, 46].

In conclusion, we report the development of an iELISA based on the HEV genotype 3 recombinant capsid protein and found a high prevalence of antibodies to HEV amongst blood donors suggesting that the region might be endemic to HEV. Even though the prevalence of HEV RNA is low in blood samples from blood banks [18] our data points to the need to investigate the presence of viral RNA in samples with high titer aiming to minimize the possibility of horizontal transmission that could be harmful to immunocompromised patients.
